# Protocol for Cilostazol Stroke Prevention Study for Antiplatelet Combination (CSPS.com): a randomized, open-label, parallel-group trial

**DOI:** 10.1111/ijs.12420

**Published:** 2014-12-08

**Authors:** Kazunori Toyoda, Shinichiro Uchiyama, Haruhiko Hoshino, Kazumi Kimura, Hideki Origasa, Hiroaki Naritomi, Kazuo Minematsu, Takenori Yamaguchi, CSPS.com Study Investigators

**Affiliations:** 1 Department of Cerebrovascular Medicine, National Cerebral and Cardiovascular Center5-7-1, Fujishirodai, Suita, Osaka, 565-8565, Japan; 2 Clinical Research Center for Medicine, International University of Health and Welfare, Center for Brain and Cerebral Vessels, Sanno Hospital and Sanno Medical Center8-10-16, Akasaka, Minato-ku, Tokyo, 107-0052, Japan; 3 Department of Neurology, Tokyo Saiseikai Central Hospital1-4-17 Mita, Minato-ku, Tokyo, 108-0073, Japan; 4 Department of Neurological Science, Nippon Medical School Graduate School of Medicine1-1-5, Sendagi, Binkyou-ku, Tokyo, Japan; 5 Division of Biostatistics and Clinical Epidemiology, University of Toyama2630 Sugitani, Toyama, Toyama, 930-0194, Japan; 6 Department of Neurology, Senri Chuo Hospital1-4-3, Shinsenri Higashimachi, Toyonaka, Osaka, 560-0082, Japan

**Keywords:** aspirin, clopidogrel, cilostazol, cerebral infarction, clinical trial, dual antiplatelet therapy, ischemic stroke, stroke prevention

## Abstract

**Rationale and aims:**

Monotherapy with antiplatelet agents is only modestly effective in secondary prevention of ischemic stroke (IS), particularly in patients with multiple risk factors such as cervicocephalic arterial stenosis, diabetes, and hypertension. While dual antiplatelet therapy (DAPT) with aspirin and clopidogrel reduced IS recurrence, particularly in the early stages after IS, it increased the risk of bleeding. Compared with aspirin, cilostazol prevented IS recurrence without increasing the incidence of serious bleeds. In patients with intracranial arterial stenosis, no significant increase in bleeding events was observed for DAPT with cilostazol and aspirin, compared to that for aspirin monotherapy. DAPT involving cilostazol may therefore be safer than conventional DAPT. These findings prompted us to conduct the Cilostazol Stroke Prevention Study for Antiplatelet Combination (CSPS.com; ClinicalTrials.gov identifier: NCT01995370) to evaluate the safety and efficacy of DAPT involving cilostazol for secondary IS prevention, in comparison with that of antiplatelet monotherapy.

**Design:**

The CSPS.com is a multicenter, randomized, open-label, parallel-group trial. A total of 4000 high-risk patients with noncardioembolic IS will be randomized 8–180 days after onset to receive aspirin or clopidogrel monotherapy, or DAPT with cilostazol and aspirin or clopidogrel for at least one-year.

**Study outcomes:**

The primary outcome is IS recurrence. Secondary outcomes are composite occurrences of any stroke, death from any cause, myocardial infarction, vascular death, and other vascular events.

**Discussion:**

The CSPS.com is expected to provide evidence indicating whether secondary IS prevention in high-risk patients can be improved by using DAPT involving cilostazol.

## Introduction and rationale

Antiplatelet therapy is a standard strategy for prevention of recurrent noncardioembolic stroke, in addition to lifestyle modifications and risk factor management. A previous meta-analysis found that conventional antiplatelet agents reduced the relative risk for stroke by about 20% in patients with a history of ischemic stroke (IS) or transient ischemic attack (TIA) 1. However, more effective antiplatelet therapy is needed in patients with intracranial arterial stenosis, carotid stenosis, or multiple vascular risk factors, because these conditions are associated with a higher risk of recurrence 2–6.

Since antiplatelet agents act through a range of mechanisms, the combination of dual agents can produce more effective stroke prevention. For example, the combination of aspirin with extended-release dipyridamole showed a superior effect on the prevention of recurrent IS, compared with aspirin alone 7,8. However, the efficacy of this combination could not be verified in a double-blind trial in Japan 9, and dipyridamole remains unapproved in Japan for prophylaxis of IS recurrence. Although relatively short-term combinations of aspirin and clopidogrel seem to prevent IS recurrence in patients with IS/TIA more effectively than aspirin alone 10–13, long-term usage is not generally recommended because it increased the incidence of serious hemorrhage and produced little reduction in the incidence of vascular events 14–16.

In the initial Cilostazol Stroke Prevention Study (CSPS) 17, this phosphodiesterase 3 inhibitor was shown to decrease IS recurrence without increasing serious bleeding, as compared with placebo. Cilostazol also decreased stroke [IS, intracerebral hemorrhage (ICH), or subarachnoid hemorrhage (SAH)] and halved serious bleeding as compared with aspirin in the second CSPS (CSPS 2) 18. In addition, a combination of cilostazol with aspirin for IS patients with intracranial arterial stenosis was investigated in three small studies 19–21; the Trial Of Cilostazol in Symptomatic intracranial arterial Stenosis (TOSS) 19 showed reduced stenosis progression, and all three studies found relatively few bleeding events, as compared with aspirin alone or aspirin plus clopidogrel.

In patients with peripheral arterial disease, the addition of cilostazol to a therapy with aspirin and/or clopidogrel did not increase bleeding times, compared with either agent alone 22. In another study of patients with peripheral arterial disease, the incidence of hemorrhagic events was comparable in subjects receiving cilostazol and in those receiving placebo 23. In a meta-analysis of randomized controlled trials in patients with a drug-eluting stent, the incidence of hemorrhagic events in the group receiving triple antiplatelet therapy (cilostazol plus aspirin and clopidogrel) was similar to that in the group receiving dual antiplatelet therapy (DAPT) with aspirin and clopidogrel 24. Thus, the addition of cilostazol may enhance the preventive effects of classical antiplatelet agents (aspirin, clopidogrel) in high-risk IS patients, without increasing hemorrhagic risk.

Animal studies have suggested that cilostazol protected mice from cerebral bleeds by reducing matrix metalloproteinase (MMP)-9 activity, and by preventing blood-brain barrier opening by inhibiting loss of claudin-5 expression 25–27. Cilostazol also increased intracellular cAMP concentration, thereby promoting the barrier function of tight junctions in brain capillary endothelial cells. Since the cAMP-dependent induction of claudin-5 expression is implicated in the promotion of endothelial cell tight junctions, the protection of endothelial cells may be explained by the phosphodiesterase-III inhibitory activity of cilostazol.

The Cilostazol Stroke Prevention Study for Antiplatelet Combination (CSPS.com; (ClinicalTrials.gov identifier: NCT01995370) will investigate the preventive effect of DAPT including cilostazol on IS recurrence in high-risk patients with noncardioembolic IS. Patients receiving cilostazol together with aspirin or clopidogrel will be compared with those treated with aspirin or clopidogrel monotherapy.

## Methods

### Design

**Figure 1:**
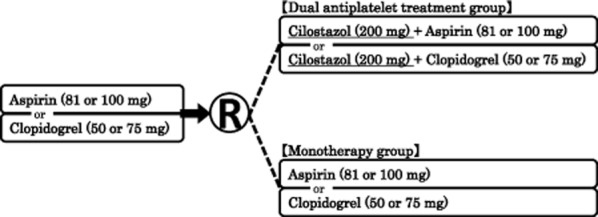
Trial design flowchart. Change in the dose of aspirin or clopidogrel will not be permitted after informed consent is obtained. Cilostazol treatment can be started with 100 mg/day, provided the dose is increased to 200 mg/day within 15 days.

The CSPS.com is a multicenter, randomized, open-label parallel-group trial. All trial centers are required to receive approval from the relevant ethics committee before initiation of the trial. Written informed consent must be obtained from each patient before enrollment. This trial is registered with ClinicalTrials.gov (NCT01995370) and the UMIN Clinical Trials Registry (000012180). Figure 1 shows a flowchart of the trial design. Table 1 shows the schedule of observations, examinations, and assessments.

**Table 1:** Trial schedule

Assessment	Period								
	(Date of onset)	Registration	Start date of observation	1 M	3 M	6 M	12 M	Thereafter, every 6 months	Completion of observation (*)
Informed consent	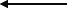	○							
Demographics	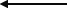	○							
Modified Rankin Scale (mRS)			○	○	○	○	○	○	○
Compliance status of trial drugs			○	○	○	○	○	○	○
Concomitant medication			○	○	○	○	○	○	○
Blood pressure			○	○	○	○	○	○	○
Head MRI	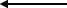	○							
Head MRA, T2*WI	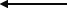	△	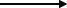						
Carotid artery imaging (US, CTA, MRA)	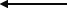	△	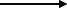						
Laboratory test (blood)	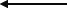	○	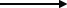						
Laboratory test (urine)	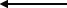	○	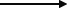						
Chest X-ray	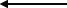	○	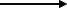						
ECG	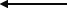	○	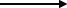						
Adverse event				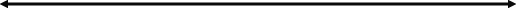					

* When patients are withdrawn from the trial, or one-year after the start of the protocol treatment in the last patient.

○, required; △, optional.

### Patient population

Patients who have developed noncardioembolic IS between 8 and 180 days before the start of the protocol treatment are the target population of the trial. Inclusion and exclusion criteria are listed in Table 2. Patients are asked to continue taking clopidogrel or aspirin alone as an antiplatelet and could not change the agent once providing informed consent. Stenosis (≥50%) of a major intracranial or extracranial artery, or two or more of the vascular risk factors listed in Table 2, is essential as an indicator of a high risk of IS recurrence.

**Table 2:** Inclusion and exclusion criteria

Inclusion criteria: 1) Clinical diagnosis of noncardioembolic IS that developed between 8 and 180 days before the start of the protocol treatment 2) A responsible lesion identified on MRI 3) Age 20–85 years 4) Taking clopidogrel or aspirin alone as antiplatelet therapy when providing informed consent 5) At least one of the following (a–c): a. ≥50% stenosis of a major intracranial artery (to the level of A2, M2, or P2) b. ≥50% stenosis of an extracranial artery (the common carotid artery, internal carotid artery, vertebral artery, brachiocephalic artery, or subclavian artery) c. Two or more of the following risk factors – Age ≥ 65 years – Diabetes mellitus – Hypertension – Peripheral arterial disease – Chronic kidney disease – History of IS (excluding the index IS for this trial) – History of ischemic heart disease – Smoking (only current smokers) 6) Considered to be able to visit the trial site for ambulatory care throughout the observation period 7) Written informed consent by the patient
Exclusion criteria: 1) High-risk sources of cardioembolism, according to the TOAST classification 2) Using any anticoagulants 3) Contraindication to MRI examination, such as claustrophobia or implanted pacemaker 4) Scheduled to undergo any surgery, such as percutaneous angioplasty, stent placement, and bypass grafting, during the trial period 5) Drug-eluting coronary stent implanted within one-year 6) History of symptomatic nontraumatic intracranial hemorrhage, any other hemorrhagic disease, bleeding predisposition, or blood clotting disorders 7) History of hypersensitivity to cilostazol 8) Congestive heart failure or uncontrolled angina pectoris 9) Thrombocytopenia (platelet count, ≤ 100,000/mm^3^) 10) Severe liver or renal dysfunction 11) Pregnant, breast-feeding, or of child-bearing potential 12) Malignant tumor requiring treatment 13) Aspirin user meeting any of the following criteria: History of hypersensitivity to aspirin or salicylic acid analogues Current peptic ulcer Aspirin-induced asthma or its history 14) Clopidogrel user with a history of hypersensitivity to clopidogrel 15) Participating in any other clinical studies 16) Unsuitable for trial enrollment, as judged by the investigator

TOAST, the Trial of Org 10172 in Acute Stroke Treatment.

### Randomization

The data center will randomize the patients to either monotherapy group or DAPT groups using a block-randomization scheme, with the research site as a randomization unit. After providing informed consent, each patient will be identified by a linkable patient identification code, and registered via a web-based registration system, along with information relevant to his or her eligibility. If the patient is considered eligible, the allocated therapy for the patient will be notified through this system. Patients and investigators will be aware of the treatment allocation.

### Treatments

Patients in the monotherapy group will receive oral aspirin (81 or 100 mg) or clopidogrel (50 or 75 mg), once daily. Clopidogrel at 50 mg is approved for older (for example ≥75 years old) or low-weight patients (≤50 kg body weight) in Japan. Patients in the DAPT group will receive oral cilostazol (100 mg, twice daily; the recommended dose in Japan) and either aspirin (81 or 100 mg) or clopidogrel (50 or 75 mg), once daily. To prevent adverse drug reactions such as headache and tachycardia, cilostazol treatment can be started at 100 mg/day, and increased to 200 mg/day within 15 days. If the patient experiences minor cilostazol-related adverse reactions including headache, palpitation, nausea, and vertigo, the trial can be continued using the reduced cilostazol dose of 100 mg/day. Change in the choice of these three antiplatelet medications will not be permitted after informed consent is obtained. Temporary suspension of the trial treatment is permitted during invasive treatment such as surgery, but cannot exceed four-weeks. Use of any concomitant treatment with any antiplatelet or anticoagulant agent (other than trial drugs) will be prohibited.

Patients will be withdrawn from the trial when they experience any cardiovascular events corresponding to the primary and secondary outcomes, or when the trial treatment has been suspended for more than four consecutive weeks.

### Primary outcome

The primary outcome is a recurrence of IS, with the symptoms lasting for at least 24 h.

### Secondary outcomes

- Any stroke (IS, ICH, and SAH)
- SAH or ICH
- IS or TIA
- Death from any cause
- Stroke, myocardial infarction (MI), or vascular death
- All vascular events, including stroke, MI, and other vascular events (e.g., aortic dissection; aortic rupture; pulmonary embolism; heart failure, angina pectoris or peripheral artery disease requiring hospitalization; revascularization of coronary artery, aorta, cephalocervical artery, and peripheral arteries).

Safety outcomes are adverse events, adverse drug reactions, and severe or life-threatening hemorrhage as defined in the Global Utilization of Streptokinase and Tissue Plasminogen Activator for Occluded Coronary Arteries (GUSTO) classification, which includes ICH and hemorrhage resulting in substantial hemodynamic compromise requiring treatment 28.

### Sample size

An annual IS recurrence rate of 4% is assumed in the monotherapy group, based on data from patients receiving aspirin monotherapy in the Japanese Aggrenox Stroke prevention versus Aspirin Programme (JASAP) study and Effective Vascular Event REduction after STroke (EVEREST) 9,29. According to Lakatos and Lan's method 30, at least 1688 patients per group would be required for the primary analysis using the log-rank test to detect a 30% relative risk reduction in the DAPT group with 80% power, a two-sided type I error of 0·05, a recruitment period of 2·5 years, and a maximum follow-up period of 3·5 years. Assuming an annual discontinuation rate of 5%, the target number of randomized patients is therefore 2000 per group, or 4000 in total. The 4000 patients will be enrolled in Japan between October 2013 and March 2016.

### Statistical analysis

Efficacy and safety analyses will be performed on the basis of the intention-to-treat (ITT) population. The treatment groups will be compared using the log-rank test. Cox proportional hazard models will be used to calculate the hazard ratio and its 95% confidence intervals for the DAPT group, compared with the monotherapy group. The hazard ratio will be adjusted for age, sex, type of IS (atherothrombotic or lacunar), and modified Rankin Scale (mRS). Annual recurrence rates will be estimated using the person-year method and the Kaplan–Meier method, and their 95% confidence intervals will be calculated using the approximate Poisson and Greenwood methods. Subgroup analyses will be performed following stratification by age, sex, antiplatelet agents (aspirin, clopidogrel), type of IS (atherothrombotic or lacunar stroke), stenosis of extracranial arteries, mRS, medical history and complications (hypertension, diabetes mellitus, dyslipidemia, coronary heart disease, peripheral arterial disease, chronic kidney disease, IS), smoking status, obesity, and microbleeds. Tests for interaction between the treatment arm and subgroup will be performed using the Cox proportional hazards model. Interim assessment of the safety and efficacy end-points will be conducted when the cumulative person-year exposure has reached 50% of the target. Early termination of the trial will be decided primarily on the basis of the Haybittle-Peto ad hoc method 31,32. Although no termination criteria are specified with regard to the safety end-points, the independent data monitoring committee (IDMC) may recommend early termination based on the incidence of adverse events within the DAPT group.

### Study organization and funding

Otsuka Pharmaceutical Co., Ltd. sponsored the trial implementation in the Japan Cardiovascular Research Foundation under an agreement between the two parties. Subject registration, data management, and statistical analysis are delegated to a contract research organization (EPS Corporation). This trial will be conducted at approximately 400 sites under the guidance of the steering committee. The enrolled patients have to pay partly their own costs for drugs prescribed in this study depending on the type of their own health insurance.

The principal investigator is Takenori Yamaguchi, who is attached to the National Cerebral and Cardiovascular Center. The final trial protocol was prepared by the protocol committee, and it will be amended, if required. Any event related to the primary and secondary end-points will be reviewed by the event review committee blindly to antiplatelet medications. The statistical analysis committee will be responsible for proposing the randomization scheme and providing expert opinions on the sample size and the analysis plan. It will be also responsible for generating the interim and final analysis plans.

The IDMC will recommend trial discontinuation/continuation or protocol amendment based on annual reviews of patient accrual, serious adverse events, incidence of adverse events, and on the interim efficacy analysis comparing the two treatment arms. The individuals who played a critical role in the planning, supervision, and conduct of this trial are listed in the end of this article.

## Discussion

Prevention of IS recurrence in high-risk patients remains an important clinical problem, since monotherapy with conventional antiplatelet agents is only modestly effective. The CSPS.com will evaluate whether DAPT including cilostazol is similarly safe and more effective in preventing IS recurrence and other vascular outcomes than aspirin or clopidogrel monotherapy in high-risk patients. This trial will resolve several clinical questions regarding preventive antiplatelet therapy for recurrent IS. First, this will be the first trial to explore the efficacy and safety of the combined use of a thienopyridine (clopidogrel) and a phosphodiesterase inhibitor (cilostazol). Second, DAPT including cilostazol may be particularly effective for Asian patients, since they frequently have intracranial atherosclerosis 33 and the TOSS showed the potential of aspirin plus cilostazol for regression of intracranial arterial stenosis 19. Since patients with stenosis (≥50%) of a major intracranial or extracranial artery can be included in the study regardless of existence or number of vascular risk factors, we expect that around half or more of the included patients will have atherothrombotic stroke. Third, DAPT including cilostazol may be safe and fit for long-term use, based on the results of previous clinical and hemostatic studies 20–25. Since Asian ethnicity is a possible risk factor for ICH 34,35, avoidance of ICH during antithrombotic therapy is essential for Asian stroke patients.

## References

[b1] Antithrombotic Trialists' Collaboration (2002). Collaborative meta-analysis of randomised trials of antiplatelet therapy for prevention of death, myocardial infarction, and stroke in high risk patients. BMJ.

[b2] Chimowitz MI, Lynn MJ, Howlett-Smith H (2005). Comparison of warfarin and aspirin for symptomatic intracranial arterial stenosis. N Engl J Med.

[b3] Kasner SE, Chimowitz MI, Lynn MJ (2006). Predictors of ischemic stroke in the territory of a symptomatic intracranial arterial stenosis. Circulation.

[b4] Nicolaides AN, Kakkos SK, Kyriacou E (2010). Asymptomatic internal carotid artery stenosis and cerebrovascular risk stratification. J Vasc Surg.

[b5] Kamouchi M, Kumagai N, Okada Y, Origasa H, Yamaguchi T, Kitazono T (2012). Risk score for predicting recurrence in patients with ischemic stroke: the Fukuoka stroke risk score for Japanese. Cerebrovasc Dis.

[b6] Diener HC, Ringleb PA, Savi P (2005). Clopidogrel for the secondary prevention of stroke. Expert Opin Pharmacother.

[b7] Diener HC, Cunha L, Forbes C, Sivenius J, Smets P, Lowenthal A (1996). European Stroke Prevention Study. 2. Dipyridamole and acetylsalicylic acid in the secondary prevention of stroke. J Neurol Sci.

[b8] The ESPRIT Study Group (2006). Aspirin plus dipyridamole versus aspirin alone after cerebral ischaemia of arterial origin (ESPRIT): randomised controlled trial. Lancet.

[b9] Uchiyama S, Ikeda Y, Urano Y, Horie Y, Yamaguchi T (2011). The Japanese aggrenox (extended-release dipyridamole plus aspirin) stroke prevention versus aspirin programme (JASAP) study: a randomized, double-blind, controlled trial. Cerebrovasc Dis.

[b10] Markus HS, Droste DW, Kaps M (2005). Dual antiplatelet therapy with clopidogrel and aspirin in symptomatic carotid stenosis evaluated using doppler embolic signal detection: the Clopidogrel and Aspirin for Reduction of Emboli in Symptomatic Carotid Stenosis (CARESS) trial. Circulation.

[b11] Kennedy J1, Hill MD, Ryckborst KJ (2007). Fast assessment of stroke and transient ischaemic attack to prevent early recurrence (FASTER): a randomised controlled pilot trial. Lancet Neurol.

[b12] Wong KS, Chen C, Fu J (2010). Clopidogrel plus aspirin versus aspirin alone for reducing embolisation in patients with acute symptomatic cerebral or carotid artery stenosis (CLAIR study): a randomised, open-label, blinded-endpoint trial. Lancet Neurol.

[b13] Wang Y, Wang Y, Zhao X (2013). Clopidogrel with aspirin in acute minor stroke or transient ischemic attack. N Engl J Med.

[b14] Diener HC, Bogousslavsky J, Brass LM (2004). Aspirin and clopidogrel compared with clopidogrel alone after recent ischaemic stroke or transient ischaemic attack in high-risk patients (MATCH): randomised, double-blind, placebo-controlled trial. Lancet.

[b15] Bhatt DL, Fox KA, Hacke W (2006). Clopidogrel and aspirin versus aspirin alone for the prevention of atherothrombotic events. N Engl J Med.

[b16] The SPS 3 Investigators (2012). Effects of clopidogrel added to aspirin in patients with recent lacunar stroke. N Engl J Med.

[b17] Gotoh F, Tohgi H, Hirai S (2000). Cilostazol stroke prevention study: a placebo-controlled double-blind trial for secondary prevention of cerebral infarction. J Stroke Cerebrovasc Dis.

[b18] Shinohara Y, Katayama Y, Uchiyama S (2010). Cilostazol for prevention of secondary stroke (CSPS 2): an aspirin-controlled, double-blind, randomised non-inferiority trial. Lancet Neurol.

[b19] Kwon SU, Cho YJ, Koo JS (2005). Cilostazol prevents the progression of the symptomatic intracranial arterial stenosis: the multicenter double-blind placebo-controlled trial of cilostazol in symptomatic intracranial arterial stenosis. Stroke.

[b20] Kwon SU, Hong KS, Kang DW (2011). Efficacy and safety of combination antiplatelet therapies in patients with symptomatic intracranial atherosclerotic stenosis. Stroke.

[b21] The CATHARSIS Study Group (2013). Final Results of Cilostazol-Aspirin Therapy Against Recurrent Stroke with Intracranial artery Stenosis (CATHARSIS). Stroke.

[b22] Wilhite DB, Comerota AJ, Schmieder FA, Throm RC, Gaughan JP, Rao AK (2003). Managing PAD with multiple platelet inhibitors: the effect of combination therapy on bleeding time. J Vasc Surg.

[b23] Hiatt WR, Money SR, Brass EP (2008). Long-term safety of cilostazol in patients with peripheral artery disease: the CASTLE study (Cilostazol: a study in long-term effects). J Vasc Surg.

[b24] Sakurai R, Koo BK, Kaneda H, Bonneau HN, Nagai R (2013). Cilostazol added to aspirin and clopidogrel reduces revascularization without increases in major adverse events in patients with drug-eluting stents: a meta-analysis of randomized controlled trials. Int J Cardiol.

[b25] Ishiguro M, Mishiro K, Fujiwara Y (2010). Phosphodiesterase-III inhibitor prevents hemorrhagic transformation induced by focal cerebral ischemia in mice treated with tPA. PLoS ONE.

[b26] Hase Y, Okamoto Y, Fujita Y (2012). Cilostazol, a phosphodiesterase inhibitor, prevents no-reflow and hemorrhage in mice with focal cerebral ischemia. Exp Neurol.

[b27] Kasahara Y, Nakagomi T, Matsuyama T, Stern D, Taguchi A (2012). Cilostazol reduces the risk of hemorrhagic infarction after administration of tissue-type plasminogen activator in a murine stroke model. Stroke.

[b28] The GUSTO investigators (1993). An international randomized trial comparing four thrombolytic strategies for acute myocardial infarction. N Engl J Med.

[b29] Suzuki N, Sato M, Houkin K (2012). One-year atherothrombotic vascular events rates in outpatients with recent non-cardioembolic ischemic stroke: the EVEREST (Effective Vascular Event REduction after STroke) registry. J Stroke Cerebrovasc Dis.

[b30] Lakatos E, Lan KK (1992). A comparison of sample size methods for the logrank statistic. Stat Med.

[b31] Haybittle JL (1971). Repeated assessment of results in clinical trials of cancer treatment. Br J Radiol.

[b32] Peto R, Pike MC, Armitage P (1976). Design and analysis of randomized clinical trials requiring prolonged observation of each patient. I. Introduction and design. Br J Cancer.

[b33] Gorelick PB, Wong KS, Bae HJ, Pandey DK (2008). Large artery intracranial occlusive disease: a large worldwide burden but a relatively neglected frontier. Stroke.

[b34] Shen AY, Yao JF, Brar SS, Jorgensen MB, Chen W (2007). Racial/ethnic differences in the risk of intracranial hemorrhage among patients with atrial fibrillation. J Am Coll Cardiol.

[b35] van Asch CJ, Luitse MJ, Rinkel GJ, van der Tweel I, Algra A, Klijn CJ (2010). Incidence, case fatality, and functional outcome of intracerebral haemorrhage over time, according to age, sex, and ethnic origin: a systematic review and meta-analysis. Lancet Neurol.

